# Factors influencing implementation of point-of-care tests for maternal and newborn screening and diagnosis in low-resource settings: a systematic review

**DOI:** 10.1186/s44263-026-00261-2

**Published:** 2026-04-11

**Authors:** Natalie Grace Shaetonhodi, Lindsey de Vos, Peya Brock, Dvora Joseph Davey, Alex de Voux, Andrew Medina-Marino

**Affiliations:** 1 Division of Epidemiology and Biostatistics, School of Public Health, University of Cape Town, Cape Town, South Africa; 2 Desmond Tutu Health Foundation, Cape Town, South Africa; 3 Department of Research, Impact Tank Analysis Foundation, Windhoek, Namibia; 4 Division of Infectious Diseases, David Geffen School of Medicine, University of California Los Angeles, Los Angeles, CA USA; 5 Perelman School of Medicine, University of Pennsylvania, Philadelphia, PA USA; 6 Division of Molecular Biology and Human Genetics, Department of Biomedical Sciences, Faculty of Health Sciences, Stellenbosch University, Stellenbosch, South Africa

**Keywords:** Point-of-care testing, Maternal health, Newborn health, Implementation science, HIV, Syphilis, Health systems, CFIR, Antenatal care, Postnatal care

## Abstract

**Background:**

Despite the potential of point-of-care (POC) tests to improve maternal and newborn health, implementation in low- and middle-income countries remains inconsistent. POC testing enables rapid, decentralized screening, but barriers across health system, facility, and individual levels may undermine uptake. This review synthesizes evidence on factors influencing the adoption, implementation, and sustainability of POC tests within maternal-child health programmes in low-resourced settings.

**Methods:**

A comprehensive search was performed across major bibliographic databases and grey literature sources using Boolean combinations of terms related to POC testing, maternal and newborn health, implementation, and low-resourced settings. Studies were eligible if they reported on adoption, implementation, or sustainability of POC tests for maternal or infant screening or diagnosis in low-resourced contexts. Data were extracted using a structured form capturing study characteristics, test types, and implementation determinants. Thematic analysis combined deductive coding using the Consolidated Framework for Implementation Research (CFIR) domains with inductive identification of cross-cutting themes. Methodological quality was appraised using Joanna Briggs Institute critical appraisal tools.

**Results:**

Forty studies from 19 low-resourced settings were included, with most evaluating POC testing for Human Immunodeficiency Virus (HIV), syphilis, and early infant HIV diagnosis, highlighting limited implementation evidence for other essential maternal and newborn diagnostics. Key facilitators of implementation included rapid turnaround time, portability, ease of use, perceived clinical benefit, supportive policy environments, and compatibility with existing workflows. Task-shifting, bundled testing, and integration with routine maternal-child health services supported sustainability. However, common barriers included weak supply chains, vertical funding, limited political will, training gaps, workforce shortages, and fragmented programme delivery. Sociocultural barriers, such as stigma and limited decision-making power among pregnant women, also constrained uptake.

**Conclusions:**

Effective implementation of POC tests in low-resourced maternal-child health settings requires system-adapted and people-centred approaches. Strengthened domestic ownership, cross-sector coordination, and integrated service delivery, will be critical to ensure equitable access and achieve global targets, including elimination of vertical transmission of HIV, syphilis, and hepatitis B. Expanded implementation research across a broader range of essential POC diagnostics and diverse geographic and infrastructure contexts is needed.

**Systematic review registration:**

PROSPERO CRD420251008480.

**Supplementary Information:**

The online version contains supplementary material available at 10.1186/s44263-026-00261-2.

## Background

Improving maternal and neonatal health remains a major challenge in low- and middle-income countries (LMICs), where high rates of preventable morbidity and mortality continue to impede progress toward the Sustainable Development Goals (SDG) [1–4]. In 2023, an estimated 287,000 maternal deaths occurred globally, with a maternal mortality rate (MMR) of 197 per 100,000 live births [5], nearly three times the SDG 3.1 target of 70 deaths per 100,000 by 2030. Over 95% of maternal deaths occurred in LMICs, with sub-Saharan Africa alone accounting for 70% [5].

Child survival disparities are similarly stark. More than 65% of the world’s under-fives live in LMICs, where mortality rates are highest. In 2023, sub-Saharan Africa’s neonatal mortality rate was 26 deaths per 1000 live births—a 34% decline since 2000, yet still more than twice the SDG 3.2 target of 12 per 1000 by 2030 [6]. Nearly 40% of under-five deaths are due to neonatal causes, such as preterm birth, birth complications, and congenital anomalies [6].

Early disease screening, detection, and management during antenatal care (ANC) are essential for identifying conditions such as HIV, syphilis and other sexually transmitted infections (STI), hepatitis B virus (HBV), malaria, and anemia, which together account for a significant proportion of preventable maternal and neonatal morbidity [7–11]. Timely antenatal screening is also a cornerstone of the World Health Organization (WHO) Triple Elimination Initiative of HIV, syphilis and HBV [12]. In 2022, an estimated 700,000 congenital syphilis cases and 390,000 syphilis-related adverse birth outcomes occurred, over half among women who were not screened during ANC [13]. Despite WHO recommendations for early hepatitis B screening among all pregnant women in settings with an HBV prevalence of 2% or higher [14], substantial gaps in antenatal screening persist [15, 16]. In the WHO African Region alone, an estimated 172,000 infants acquired HBV through vertical transmission in 2022 [17]. Pregnancy and breastfeeding are also periods of heightened vulnerability to HIV [18, 19]. New maternal HIV infections account for roughly one-third of cases of vertical transmission [20], underscoring timely detection and treatment among pregnant and breastfeeding women as a critical global health priority.

Many of these conditions are asymptomatic or present with non-specific symptoms, requiring aetiological screening and diagnosis for effective intervention [21–24]. However, access to quality screening and diagnostic tests remain limited in LMICs [25–27]. Laboratory services are often centralized, resulting in long turnaround times and creating barriers to care for women attending lower-level or rural facilities [28–30]. These challenges, compounded by poor retention in health services during pregnancy and breastfeeding [31–36], lead to delayed or missed opportunities for treatment and contribute to poor maternal and neonatal outcomes [8, 37–40].

Point-of-care (POC) tests provide rapid, decentralized screening and diagnosis in primary health clinics, health posts, communities, and homes, requiring minimal infrastructure and training. Rapid tests, including self-tests, have enhanced detection and treatment of HIV, syphilis, HBV, and malaria during ANC, helping reduce vertical transmission [41–43]. Aetiological POC screening for curable STIs has been shown to improve initiation of pre-exposure prophylaxis (PrEP) among pregnant women attending ANC [44], and modelling studies suggest it could also reduce maternal HIV acquisition by about 10% during pregnancy and 4.7% during breastfeeding, contributing to a further 9% decline in vertical HIV transmission [45]. Other POC tools, such as obstetric ultrasound (O-POCUS), haemoglobin testing, and blood typing, have demonstrated feasibility [46–51] and cost-effectiveness [52–57] in low-resource ANC settings, offering critical opportunities for the timely management of high-risk maternal and fetal conditions.

Despite their potential, integration of POC tests into routine maternal-child health services remains limited in many LMICs [58] While previous reviews have examined factors shaping POC implementation in low-resource settings, many have been disease- or test-specific—such as focusing on dual HIV/syphilis rapid tests, STI screening, or other vertical programmes—or have been conducted outside maternal and neonatal health [59–62]. These narrower scopes limit understanding of the comparative implications for adoption, integration, and long-term sustainability within antenatal and postnatal care, where screening must be delivered alongside multiple, interdependent services and where limited resources must be prioritized.

This systematic review aimed to provide cross-cutting insight into the factors that directly influence POC test uptake and usage in maternal–child health programmes by (1) identifying factors influencing the adoption, implementation, and sustainability of POC tests for maternal and infant screening and/or diagnosis in resource-limited settings, and (2) synthesizing findings using the five domains of the Consolidated Framework for Implementation Research (CFIR) as a conceptual guide [63].

## Methods

### Eligibility criteria

Eligibility criteria were defined using the PICOS framework (Population, Intervention, Comparator, Outcomes, Study design), with detailed criteria provided in Supplementary material 1. Studies were included if they evaluated the pre, pilot, or post stages of implementation of POC tests for screening or diagnosis of infectious disease or non-infectious conditions among pregnant women or infants within LMIC. Eligible designs included observational, randomized, quasi-experimental, modelling, qualitative, mixed-methods studies, meta-analyses, implementation or program evaluations, field or facility assessments, and quality improvement studies. Grey literature, including WHO reports and conference abstracts, were also reviewed. Systematic and scoping reviews were excluded.

The population of interest encompassed pregnant women, infants, mother–baby dyads, healthcare providers, facility managers, and policymakers involved in POC screening or diagnostic delivery, uptake, or decision-making. Interventions involved tools used to screen or diagnose infectious diseases or conditions among pregnant women, neonates, and infants at the point-of-care. We included all POC tests regardless of turnaround time to enable us to evaluate turnaround time as an influencing factor. Studies were excluded if they focused exclusively on laboratory-based interventions or assessed only diagnostic accuracy without examining implementation factors. Because this review examined implementation studies, no comparator was required. Eligible outcomes included reported factors influencing adoption, implementation, or sustainability of POC tests within maternal-child health screening and/or diagnostic programmes.

### Key definitions

Low-resourced settings were defined as antenatal or postnatal service-delivery points in LMIC.

Point-of-care (POC) tests were defined as screening or diagnostic tools performed at or near the site of patient care without requiring centralized laboratory infrastructure. Tests included both rapid and instrument-based platforms used in antenatal and postnatal care and varied in complexity, sample type, equipment needs, and turnaround time. Detailed definitions of all test types are provided in Supplementary material 1: Text S1.

### Search strategy

A comprehensive literature search was conducted in PubMed/MEDLINE, Scopus, and Google Scholar using a combination of MeSH terms and free-text keywords related to antenatal care, newborn care, point-of-care and rapid testing, and implementation. Boolean operators and truncation were applied to optimize sensitivity and specificity. The search was restricted to studies published between January 2015 and April 2025 to ensure relevance to contemporary POC tests and implementation contexts. The search strategy was applied for peer-reviewed and grey literature sources. Due to differences in database functionality, the strategy was adapted for Google Scholar by using simplified keyword strings. The full search strategy is available in Supplementary material 1: Text S2.

### Screening strategy

**Fig. 1:**
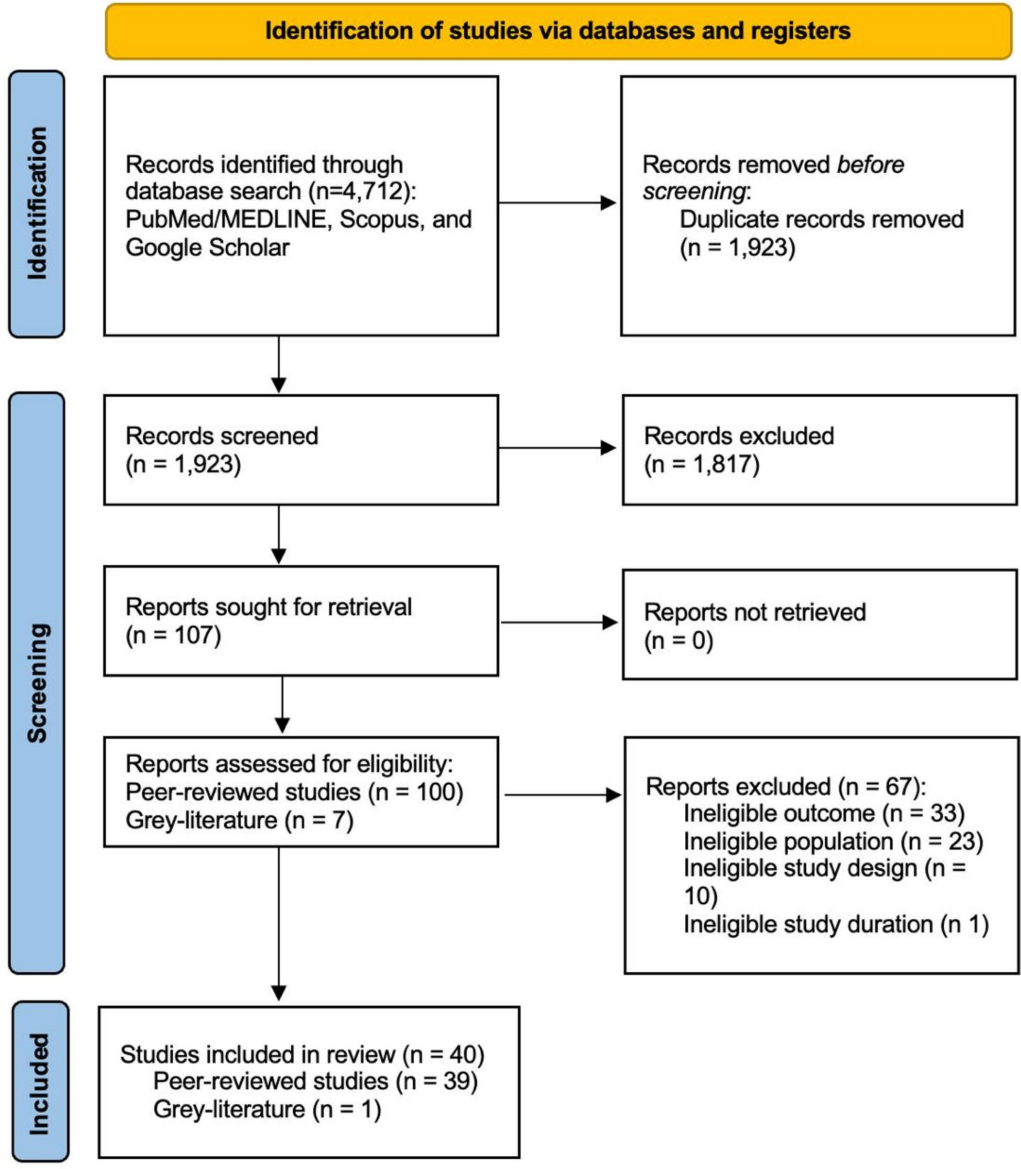
PRISMA flow diagram of systematic review study selection

Study selection was conducted in four stages. First, titles were screened for relevance by NGS, and relevant records were imported into Zotero (Corporation for Digital Scholarship, Vienna, VA, USA) (version 6.0.37). Second, duplicates were identified and removed. Third, abstracts were independently screened by NGS and LdV. Finally, full texts of potentially eligible studies were reviewed against the inclusion criteria. Studies not meeting criteria were excluded at each stage. Rayyan (Rayyan Systems, Inc., Cambridge, MA, USA) [64] a web-based systematic review platform, was used for management of abstract and full text reviews. Discrepancies were resolved through discussion, with a third reviewer (AdV) available for arbitration. The selection process was documented with the Preferred Reporting Items for Systematic Reviews and Meta-Analyses (PRISMA) flow diagram [65] (Fig. 1).

### Quality appraisal

We conducted a quality appraisal of included studies using the respective checklists from the Joanna Briggs Institute (JBI) critical appraisal tools [66], selected according to study design. Each study was independently assessed by NGS using the full set of items from the relevant JBI checklist, with responses recorded as “yes,” “no,” or “unclear.” One point was awarded for each “yes” response, while “no” response received zero points. A total proportion was calculated by dividing the number of “yes” responses by the total number of checklist items. Based on these scores, studies were categorized as having low risk of bias (≥ 80%), moderate risk of bias (50–79%), or high risk of bias (< 50%). Mixed methods studies [67–73] were evaluated using the appropriate JBI checklist aligned to the primary outcome of the study (i.e., qualitative or quantitative by design). We assessed risk of bias from missing results by examining studies for selective reporting and incomplete data, considering overall reporting completeness across studies. Confidence in each CFIR domain was based on the consistency of findings, diversity of settings and designs, and depth of qualitative evidence. Quality appraisal results were used to support sensitivity analyses and inform the narrative synthesis of findings but were not used as criteria for inclusion or exclusion.

### Conceptual framework

CFIR [63] was used to guide this review due to its comprehensive, theory-informed structure for identifying key implementation determinants. CFIR includes five domains: (1) Intervention, (2) Outer Setting, (3) Inner Setting, (4) Individuals, and (5) Process. CFIR has been widely applied across disciplines such as epidemiology, clinical care, and behavioural health to evaluate interventions at all stages of implementation, including before, during, and after rollout [74]. CFIR is well-suited to synthesize findings across heterogeneous maternal-child health settings in low-resource environments. It was chosen for this review to enable a nuanced understanding of multilevel implementation dynamics which support the development of actionable insights to inform the integration, scale-up, and sustainability of POC tests.

### Data extraction, analysis, and management

We used a structured Excel (Microsoft Corporation, Redmond, WA, USA) spreadsheet to extract key information from each study, including author, year, country, setting, population, and findings aligned with the five CFIR domains. Outcomes of interest included determinants of adoption (defined as factors influencing initial uptake and acceptance of POC testing by providers and clients), implementation (defined as factors affecting integration, fidelity, and quality of POC testing within antenatal care workflows), and sustainability (defined as factors enabling continued use and scale-up of POC testing over time within routine health services). All reported results relevant to these outcome domains were extracted. Excerpts from each included study were coded by NGS and thematically organized in the data extraction table across the five CFIR domains: Intervention Characteristics, Outer Setting, Inner Setting, Characteristics of Individuals, and Process. PB reviewed all codes for accuracy and alignment with the assigned domain. Discrepancies were discussed with an independent reviewer (AdV) available for arbitration. Data from the extraction table and coded excerpts were tabulated to generate descriptive summaries of study characteristics.

Thematic analysis was conducted using both inductive and deductive approaches. A deductive coding structure, based on the five CFIR domains, guided the initial organization of extracted data. Within this framework, inductive analysis was used to identify emergent themes and context-specific determinants that were not predefined in CFIR. Codes were then examined across all studies to identify overarching patterns and subgroup analyses were conducted by disease area (e.g., syphilis, HIV, malaria) to explore variation in factors influencing adoption, implementation, and sustainability. The complete data extraction table is provided in Supplementary material 2: Data extraction table.

### Protocol and registration

This systematic review was conducted in accordance with the PRISMA Statement [65]. The completed PRISMA checklist is available in Supplementary material 3: PRISMA 2020 Checklist. The review protocol, including the comprehensive search strategy, was prospectively registered on the International Prospective Register of Systematic Reviews (PROSPERO; Registration Number: CRD420251008480).

## Results

### Study characteristics

The initial search was conducted on January 12th, 2025, and yielded 4712 articles. Of these articles, 40 studies met inclusion criteria, 39 peer-reviewed and 1 grey literature (Fig. 1).

Of those 40 studies, 19 (48%) were qualitative [75–92], 14 (35%) were quantitative [71, 93–105], and 6 (15%) were mixed methods [67–73]. Studies were conducted in Africa (*n* = 34, 85%), Asia (*n* = 4, 10%), and South America (*n* = 2, 5%). Within Africa, Ghana was the most represented country (*n* = 9) [75–78, 95, 98–101], followed by Kenya (*n* = 6) [81, 83, 84, 91, 104, 105]. In Asia–Pacific, studies were conducted in Nepal (*n* = 2) [79, 93], Myanmar (*n* = 1) [68], and Papua New Guinea (*n* = 1) [89]. In South America, studies were conducted in Guatemala (*n *= 1) [102] and Columbia (*n* = 1) [71]. Most studies focused on rapid syphilis testing (*n* = 10, 25%) [67, 71, 72, 75, 87, 94, 95, 101], followed by POC testing for HIV early infant diagnosis (EID) or birth testing (*n *= 7, 18%) [80, 83, 85, 86, 89, 90, 103]. The target population(s) of the studies included pregnant women, newborns, or caregivers (67.5%, *n* = 27) [67–73, 76, 78–80, 83–86, 91, 92, 94–97, 101–106], health care workers (60%, *n* = 24) [67–69, 72, 73, 75–82, 84–86, 88–93, 98, 104], facility or district-level leaders (27.5%, *n *= 11) [69, 75, 82, 86, 88–90, 92, 93, 99, 100], and national or sub-national policy-makers (22.5%, *n* = 9) [67, 70, 79, 80, 82, 88, 89, 91, 107]. Most studies (*n* = 18, 45%) were conducted after national or targeted rollout of POC test integration [69, 70, 75–77, 79, 81, 86–88, 90, 93–95, 99–101], followed by 12 studies (30%) conducted during pilot stage to inform national rollout [67, 68, 71–73, 80, 82, 84, 85, 102, 104, 105]. Nine studies (23%) were conducted prior to implementation to assess feasibility, acceptability, and formative resource needs [78, 83, 89, 91, 92, 96, 97, 103, 106] (Table 1). Point-of-care test types and reported technical characteristics included in systematically reviewed studies are described in Table 2. Two studies in Ghana and Nepal included insights from both ANC and non-ANC settings [77, 93].

**Table 1:** Characteristics of systematically reviewed studies (*n* = 40)

Study characteristics	Number of studies	Percent
Region		
Africa	**34**	**85%**
Burkina Faso	1	3%
Eswatini	1	3%
Ghana	9	23%
Kenya	6	15%
Malawi	1	3%
Mozambique	1	3%
South Africa	3	8%
Tanzania	2	5%
Zambia	2	5%
Zimbabwe	2	5%
Cameroon, Côte d'Ivoire, Eswatini, Kenya, Lesotho, Mozambique, Rwanda, and Zimbabwe	1	3%
Central African Republic, Ghana, Madagascar, Mozambique, Tanzania, and Zambia	1	3%
Democratic Republic of the Congo and Zambia	2	5%
Tanzania and Uganda	1	3%
Asia	**4**	**10%**
Nepal	2	5%
Myanmar	1	3%
Papua New Guinea	1	3%
South America	**2**	**5%**
Columbia	1	3%
Guatemala	1	3%
Publication date		
2015–2019	25	63%
2020–2025	15	38%
Study design		
Qualitative	19	48%
Mixed methods	6	15%
Quasi-experimental	6	15%
Cross-sectional	6	15%
Case-control	1	3%
Cluster randomized controlled trial	1	3%
Policy and practice workshop review	1	3%
POC intervention		
Maternal HIV rapid tests	2	5%
Maternal POC viral load tests	1	3%
Maternal POC viral load and EID tests	1	3%
POC HIV EID or birth testing	7	18%
Rapid syphilis tests (RST)	10	25%
HIV/syphilis dual rapid test	1	3%
Malaria rapid diagnostic test (mRDT)	1	3%
Glucose-6-phosphate dehydrogenase (G6PD) deficiency test	1	3%
STI screening (GeneXpert CT/NG and TV assays and NG-LFA)	2	5%
Obstetric point-of-care ultrasound (O-POCUS)	1	3%
Maternal HIV and RST	2	5%
Malaria RDT and anemia screening	1	3%
Blood group and rhesus type tests	1	3%
Bundled PMTCT screening (HIV, syphilis, and Hepatitis B)	2	5%
HIV rapid tests, RST, and mRDT	1	3%
HIV rapid tests, RST, and anemia screening	2	5%
Bundled screening for HIV, syphilis, malaria, and anemia	3	8%
Clinical decision support system (CDSS) for pre-eclampsia, gestational diabetes, anemia POC screening	1	3%
Malaria, HIV, urine pregnancy, blood pressure monitoring, syphilis, and hemoglobin, and other essential POC screening tests	1	3%
Target population		
Pregnant women, caregivers, HIV-exposed infants	27	67.5%*
Front-line health care workers (nurses, midwives, physician assistants, physicians, community health workers, and laboratory staff)	24	60%*
Facility or district-level leaders, including programme managers and administrators	11	27.5%*
National or sub-national policy-makers	9	22.5%*
Stage of national implementation at time of study		
Pre	9	23%
Pilot	12	31%
Post	18	46%
Varying stages by country	1	3%
Risk of bias		
Low risk	31	76%
Moderate risk	6	15%
High risk	1	3%
Quality assessment not applicable	2	5%

*EID *early infant diagnosis, *G6PD* glucose-6-phosphate dehydrogenase deficiency, *mRDT* malaria rapid diagnostic test, *NG-LFA* Neisseria gonorrhoeae lateral flow assay, *O-POCUS* Obstetric point-of-care ultrasound, *PMTCT* Prevention of mother-to-child transmission, *RST* rapid syphilis test

^*^Includes studies with multiple target groups, as such percentages are over 100%

**Table 2:** Point-of-care test types and associated operational characteristics reported in systematically reviewed studies

POC test type^a^	Reported manufacturer/brand names and associated studies	Antenatal or postnatal application	Reported technical characteristics	Reported equipment needs	Reported turnaround time
Rapid syphilis test	• Alere Determine Syphilis TP test; (Alere International, UK) [67, 75, 94] • SD Bioline Syphilis 3.0 test; (Standard Diagnostic, Yongin, Korea) [73, 94, 102, 105] • Not specified [70–72, 79, 88, 92, 95, 101]	Screening for all pregnant women at the first ANC visit (subsequent visits based on national test guidelines)	• Finger prick specimen collection requiring very small volume of blood, compared with venous blood for RPR • Procedure is similar to HIV rapid testing • Test cartridges are single-use and disposable	• RST test kit (single-use card/cassette + buffer), Fingerpick lancet and capillary pipette • Treatment (Benzathine penicillin G/erythromycin) • No electricity, refrigeration, or laboratory equipment needed,	15 min to 1 h (depending on resource availability and facility type)
HIV birth testing and early infant diagnosis	• Xpert HIV-1 Qualitative assay (Cepheid, Sunnyvale, CA, USA) [83, 89, 97, 103] • Abbott m-PIMA HIV-1/2 detect [83, 85, 97] • Not specified [80, 86, 90]	Birth testing: HIV-exposed infants at birth (timing based on national guidelines) Early infant diagnosis: HIV-exposed infants at 4–6 weeks, 9 months, and 18 months (based on national guidelines)	• Heel-prick infant blood sample • Single-use cartridge-based. Cartridges can be stored for up to 72 h • Portable, battery-operated device • On-screen prompts • Single-use test cartridges • Testing conducted in community-based settings by lower-level research assistants • Sensitive to high ambient temperatures	• Cartridges • Microvettes, venipuncture supplies, pipettes • Stable electricity • Private counselling space	90 min to 2 h
Malaria rapid diagnostic test	• CareStart™ Malaria HRP2 Pf (AccessBio, USA) [105] • Not specified [76, 77, 98]	Pregnant women with suspected malaria	• Detects falciparum species; negative results did not exclude non-falciparum malaria • Finger prick sampling • Lines on reader indicate positivity • Portable	• Storage area • Job aids	2–45 min
HIV rapid test	• HIV (1 + 2) Antibody Colloidal Gold (KHB, China) [105] • Determine HIV-1/2 rapid test (Abbott Laboratories, Tokyo, Japan) [102] • Not specified [68–70, 79, 88, 93, 98]	All pregnant women at first ANC (retesting as per national guidelines)	• Finger prick sampling • Delivered at mobile outreach points and community-based settings	• Rapid test kits, lancets, gloves, alcohol swabs • Private counselling space	15 min
Haemoglobin test	• HemoCue® Hb 201 + (HemoCue AB, Sweden) [70, 73, 105] • Haemoglobin Colour Scale (HCS) [76] • Not specified [98]	Routine anemia screening throughout pregnancy	• Finger prick sampling • Compares the color of a treated sample against a graded colour scale to estimate Hb concentration (HCS) • Digital haemoglobin concentration in g/dL, results read via the HemoCue® machine (HemoCue)	• HemoCue® Hb 201 + analyzer and microcuvettes • HCS cards • Special reagent paper (HCS) • No electricity, laboratory equipment, or complex processing required • Timer	Immediate
*Chlamydia trachomatis, Neisseria gonorrhoeae, Trichomonas vaginalis* tests	• GeneXpert CT/NG and TV Assays (Cepheid, Sunnyvale, CA, USA) [96] • NG Lateral Flow Assay (Prototype) [106]	All pregnant women at first ANC	• Vaginal swab • Cartridge-based molecular NAAT (GeneXpert) • Multiplex assay (CT/NG) (GeneXpert) • CT/NG and TV assays run in parallel on same machine (GeneXpert) • Single-use disposable lateral flow assay (NG-LFA) • Battery-powered fluorescent europium reporter for antigen detection (NG-LFA)	• Continuous electricity supply (GeneXpert) • Cartridges (GeneXpert) • Portable fluorescence reader and batteries (NG-LFA)	60–90 min (GeneXpert) 20 min (NG-LFA)
Dual HIV/syphilis rapid test	• Chembio Dual Path Platform (DPP®) HIV/syphilis assay [82] • Not specified [107]	Pregnant women with previous negative or unknown HIV status women at first ANC (retesting based on national guidelines)	• Multiplex assay • Digital reader • Three result lines (HIV, syphilis, control) • 4 procedural steps (vs. 3 for single pathogen test) (DPP) • Requires use of two wells and two solutions: a buffer and a sample diluent (DPP) • Precise timing: 5-min test running period plus 10-min waiting period before result interpretation (DPP) • Blood is placed in a buffer/diluent bottle before adding to device (DPP)	• Buffer bottle, sample diluent, test cartridge, lancet/fingerstick, and timer/stopwatch (DPP) • No electricity required	15 min
Hepatitis B surface antigen testing	• Determine HBsAg test (Alere Inc., MA, USA) [102] • Not specified [79]	All pregnant women at first ANC	• Single-use, lateral flow rapid diagnostic test • Single finger-prick blood sample • Portable, conducted in lower-level and community settings	• Rapid test kit • Finger-prick lancets	15 min
HIV viral load	• Xpert HIV-1 Qualitative assay (Cepheid, Sunnyvale, CA, USA) [91, 97]	Pregnant and postpartum women living with HIV (timing and frequency based on national guidelines/study protocol)	• Multi-disease use: the same GeneXpert machines were concurrently used for TB and COVID-19 • Cartridge-based • Whole blood specimens • Result return to caregivers via SMS	• GeneXpert instrument for VL quantification • Single-use Xpert HIV-1 VL cartridges • Continuous electricity required	3.3 h (median) 1–2 days for near-POC
CD4 + T-cell count	• Alere PIMA CD4 Analyzer (Alere Inc., Waltham, MA, USA) [73]	Pregnant women living with HIV for clinical staging	• Cartridge-based, instrument-assisted platform	• Alere PIMA instrument	Not reported
Integrated diagnostic and clinical decision support system (CDSS)	• Bliss4Midwives (B4M) [78] (Prototype)	Routine anemia, preeclampsia, and gestational diabetes screening throughout pregnancy	• Integrated and non-invasive • Infrared finger-clip measuring hemoglobin without blood draw • Self-inflating, automated blood pressure cuff • Automated dipstick reader • Android tablet with integrated CDSS which is automatically or manually linked to device outputs and provides algorithm-based decision support • Traffic-light signalling system indicates risk category and referral urgency	• B4M device • Infrared haemoglobin finger clip • Automated BP cuff • Urine dipstick and automated dipstick reader • Android tablet	Not reported
ABO blood group and Rh (rhesus) type	• Not specified [98, 99]	All pregnant women at first ANC	• Slide agglutination test • Capillary whole blood via finger prick • Visual reading of agglutination patterns	• Not reported	Not reported
Glucose-6-phosphate dioxygenase deficiency	• Not specified [98, 100]	Pregnant women in Malaria endemic areas	• Capillary whole blood via finger prick	• Not reported	Not reported
Obstetric point of care ultrasound (O-POCUS)	• Not specified [81]	All pregnant women at routine ANC visits for screening and clinical management	• Portable • Tablet-based system with a probe • Provides real-time images • Sensitive to high temperatures	• Electricity for charging • Internet access for uploading or updating software • Ultrasound gel • Paper towels/tissue	Immediate

^a^Definitions of test types are reported in Supplementary material 1: Text S1

### Quality appraisal results

Of the 40 articles included in the review, 38 underwent quality appraisal. Two articles were excluded from appraisal: Newman Owiredu et al. [107] was a policy review based on a stakeholder workshop and not an empirical study, making it ineligible for appraisal using JBI tools. Potes et al. [71] was a conference abstract lacking sufficient methodological detail for assessment. Both were retained for contextual relevance but excluded from formal risk of bias appraisal, in accordance with systematic review best practices [108].

Overall, 82% (*n *= 31) of the appraised studies were rated as low risk of bias, with an average score of 90%. All 19 studies with primary qualitative outcomes [72, 75–92], were rated low risk of bias (average score 91%), with the most common limitation being lack of researcher reflexivity and/or theoretical positioning statements in 13 studies [72, 76, 78–82, 85, 88–92]. Among mixed-methods studies, all 6 had quantitative primary outcomes and were appraised using the appropriate JBI checklist based on quantitative design. Of the 20 studies with quantitative primary analyses—including quasi-experimental [67, 71, 73, 96, 97, 102, 103, 105], cross-sectional [68, 69, 93, 98–101, 104], cluster-randomized [94], case–control [95], multiple case study [70], and prospective test validation [106], 12 (60%) were rated low risk of bias and 6 (30%) as moderate. One quasi-experimental study was rated high risk (score 44%). Key limitations in quantitative studies included lack of adjustment for confounders [67, 98–100, 102], absence of control groups [67, 73, 102, 105], and lack of blinding [94, 95]. Across studies, selection bias was moderate due to the frequent use of convenience or consecutive sampling. The risk of bias from missing or selective reporting was generally low to moderate, as most studies adequately described their methods and findings. Overall confidence in the body of evidence was moderate to high; however, confidence in sustainability-related outcomes was lower due to the absence of longitudinal implementation studies. Full appraisal results are provided in Supplementary material 4: Quality appraisal results.

### Thematic synthesis

Across the 40 included studies, several determinants consistently influenced the adoption, implementation, and sustainability of POC testing in maternal and newborn health services. Key facilitators included ease of use, suitability for decentralized settings, perceived benefits for maternal and infant outcomes, integration with existing ANC workflows, and person-centred provider–client interactions. Common barriers included vertical financing constraints, supply chain disruptions and stockouts, low facility readiness, staffing shortages and workload pressures, inadequate training, and hesitancy stemming from concerns about test accuracy. Detailed findings for each CFIR domain are presented below and summarized in Tables 3, 4, 5, 6, and 7.

**Table 3:** Intervention characteristics influencing implementation of POC tests for maternal–infant screening and diagnosis

Reported barriers and facilitators	Associated studies and point-of-care interventions	Number of studies reporting barrier	% of included studies reporting barrier (*N* = 40)
*Operational usability and workflow integration*			
Barriers			
Long turnaround time	• HIV EID/birth testing [80, 86, 89, 97]; • Bundled screening for HIV, syphilis, malaria, and anemia; [84, 105]; • STI screening (GeneXpert CT/NG and TV) [96]; • Maternal HIV viral load [97]; • Anemia screening [76]	7	17.5%
Resource and maintenance intensive	• HIV EID/birth testing [89, 90]; • HIV RDT [68]; • O-POCUS [81]	4	10%
Complex procedures	• Dual HIV/Syphilis rapid test [82]	1	2.5%
Disruptive sample collection	• Integrated diagnostic and clinical decision support system (CDSS) for POC anemia, preeclampsia, and gestational diabetes screening [78]	1	2.5%
Facilitators			
Ease-of-use	• RST [67, 84, 87, 92], • HIV EID/birth testing [85, 90]; • mRDT [76, 84]; • Anemia screening [76, 84] • STI screening (NG-LFA) [106]	8	20%
Single finger prick sampling for multiple tests	• RST [67, 72, 75]; • Bundled screening for HIV, syphilis, malaria, and anemia [84, 105] • HIV RDT [75]; • Maternal HIV viral load PCR [97]; • Bundled PMTCT screening (HIV, syphilis, and HBV) [102];	7	17.5%
Simplified testing algorithm or sample collection	• HIV EID/birth testing [86, 89]; • RST [72];	3	7.5%
Trialability	• Bundled screening for HIV, syphilis, malaria, and anemia [84]	1	2.5%
*Design features*			
Barriers			
Difficult to interpret	• Dual HIV/syphilis rapid test [84]; • mRDT [76]; • Bundled screening for HIV, syphilis, malaria, and anemia [84]	3	7.5%
Facilitators			
Observable results and on-screen prompts	• O-POCUS [81]; • Anemia screening [76]; • HIV EID/birth testing [85]; • Bundled screening for HIV, syphilis, malaria, and anemia [84]	6	15%
Portability	• RST [87]; • mRDT [77]; • STI screening (NG-LFA) [106]; • O-POCUS [81]; • HIV EID/birth testing [85]	5	12.5%
Multi-disease testing and multiplex assays	• Dual HIV/Syphilis rapid test [82, 107]; • HIV EID/birth testing [89]; • Maternal HIV viral load [91]	4	10%
Simple interpretation	• RST [75, 87]; • Dual HIV/syphilis rapid test [107]	3	7.5%
Long battery life	• STI screening (NG-LFA) [106]; • HIV EID/birth testing [85]	2	5%
*Relative advantage*			
Barriers			
No barriers reported		0	0%
Facilitators			
Reduced wait time	• HIV EID/birth testing [80, 83, 85, 90]; • Bundled PMTCT screening (HIV, syphilis, HBV) [88]; • mRDT [88]; • STI screening (NG-LFA) [106]; • Maternal HIV viral load [97]	7	17.5%
Reduced loss-to-follow up	• HIV EID/birth testing [83, 89, 90]	3	7.5%
Reduced need for referral and follow-up	• Bundled screening for HIV, syphilis, malaria, and anemia [88]; • RST [81]; • Anemia screening [81]; • HIV, malaria, sickling, hepatitis C, blood type, and tuberculosis [98]; • O-POCUS [81];	3	7.5%
*Reliability and test performance*			
Barriers			
Test validity concerns and frequent errors	• HIV EID/birth testing [83, 97, 103]; • Maternal HIV viral load PCR [91, 97]; • STI screening (NG-LFA) [106]; • mRDT [76]; • RST [79]; • HBV screening [79];	7	17.5%
Technical issues	• HIV EID/birth testing [80, 90]; • Integrated diagnostic and clinical decision support system (CDSS) for POC anemia, preeclampsia, and gestational diabetes screening [78]; • STI screening (NG-LFA) [106]; • O-POCUS [81]	5	12.5%
Limited diagnostic scope	• mRDT [76]	1	2.5%
Facilitators			
Strong test reliability	• RST [72]; • Dual HIV/syphilis rapid test [82]	2	5%
*Cost and affordability*			
Barriers			
High costs of test equipment and maintenance	• RST [75, 92]; • mRDT [77]; • Dual HIV/syphilis rapid test [82]; • HIV EID/birth testing [89]; • Bundled PMTCT screening (HIV, syphilis, HBV) [79]	6	15%
Facilitators			
Low indirect costs to pregnant women and caregivers	• Dual HIV/syphilis rapid test [82]; • Integrated diagnostic and clinical decision support system (CDSS) for POC anemia, preeclampsia, and gestational diabetes screening [78]; • RST [72]; • HIV EID/birth testing [85]	4	10%

*CT *chlamydia trachomatis, *EID* early infant diagnosis, *HBV* hepatitis B virus, *mRDT* malaria rapid diagnostic test, *NG* Neisseria gonorrhoeae,* NG-LFA* Neisseria gonorrhoeae lateral flow assay, *O-POCUS* Obstetric Point-of-care Ultrasound, *PMTCT* Prevention of mother to child transmission, *QA/QC* Quality Assurance and Quality Control, *RST* rapid syphilis test, *RDT* Rapid Diagnostic Test; *TV* trichomonas vaginalis

**Table 4:** Outer setting factors influencing implementation of POC tests for maternal–infant screening and diagnosis

Reported barriers and facilitators	Associated studies and point-of-care interventions	Number of studies reporting factor	% of included studies reporting factor (*N* = 40)
*National policy and financing environment*			
Barriers			
Vertical donor financing	• RST [70, 87]; • mRDT [76, 77]; • Bundled PMTCT screening (HIV, syphilis, HBV) [79, 88] • Bundled screening for HIV, syphilis, malaria, and anemia [84, 105] • Anemia screening [76]; • HIV RDT [69];	9	22.5%
Low prioritization in national and sub-national policies and budgets	• RST [75]; • Bundled PMTCT screening (HIV, syphilis, HBV) [79, 102]; • Dual HIV/syphilis rapid test [82, 107]; • HIV EID/birth testing [80]	6	15%
Competing funding for diagnostics	• Bundled PMTCT screening (HIV, syphilis, HBV) [79, 88]	2	5%
Lack of regulatory and reimbursement mechanisms for private providers	• mRDT [77]	1	2.5%
Facilitators			
Supportive policies, such as free ANC services and free testing for pregnant women	• RST [67, 75, 92]; • Dual HIV/syphilis rapid test [107]; • Maternal HIV viral load [91]	5	12.5%
National and sub-national government leadership	• HIV EID/birth testing [89]; • O-POCUS [81]; • Bundled PMTCT screening (HIV, syphilis, HBV) [88]; • Dual HIV/syphilis rapid test [107]	3	7.5%
Sustainable domestic financing	• RST [67]; • Dual HIV/syphilis rapid test [82]	2	5%
Prioritization in national policies and guidelines	• Dual HIV/syphilis rapid test [82]	1	2.5%
*Stakeholder engagement*			
Barriers			
Lack of inclusion of technical and front-line stakeholders in implementation	• RST [75]; • Bundled PMTCT screening (HIV, syphilis, HBV) [88]	2	5%
Facilitators			
Broad stakeholder engagement, including donors and implementing partners	• Bundled PMTCT screening (HIV, syphilis, HBV) [88, 102]; • Dual HIV/syphilis rapid test [82]; • Maternal HIV viral load [91]; • HIV EID/birth testing [90]	4	10%
*Supply chains and procurement systems*			
Barriers			
Supply chain disruptions	• RST [70, 71, 75] • HIV RDT [68]; • Bundled PMTCT screening (HIV, syphilis, HBV) [88]; • Maternal HIV viral load [91]; • Bundled screening for HIV, syphilis, malaria, and anemia [84]	7	17.5%
Ineffective procurement and forecasting	• Bundled PMTCT screening (HIV, syphilis, HBV) [88]	1	2.5%
Facilitators			
Integrated supply chains	• Dual HIV/syphilis rapid test [82, 107]	2	5%
*Sociocultural, socioeconomic, and access to care*			
Barriers			
Stigma	• RST [72, 92]; • HIV RDT [68]; • Bundled PMTCT screening (HIV, syphilis, HBV) [79]; • HIV EID/birth testing [85]; • Bundled screening for HIV, syphilis, malaria, and anemia [84]	6	15%
Geographic barriers and late ANC attendance impacting access to POC services	• HIV RDT [68]; • RST [92]; • HIV EID/birth testing [80]; • Dual HIV/syphilis rapid test [107]; • Bundled screening for HIV, syphilis, and anemia [70]	6	15%
Discouragement from the community or household to engage in testing (low decision-making power of women)	• RST [72, 92]; • Bundled PMTCT screening (HIV, syphilis, HBV) [79, 102]; • HIV EID/birth testing [80]	4	10%
Facilitators			
Community and household outreach and support	• HIV EID/birth testing [80, 83, 86]; • Dual HIV/syphilis rapid test [107]; • Bundled PMTCT screening (HIV, syphilis, HBV) [102]	5	12.5%

*CT* chlamydia trachomatis, *EID* early infant diagnosis, *HBV* hepatitis B virus, *mRDT* malaria rapid diagnostic test, *NG* Neisseria gonorrhoeae, *NG-LFA* Neisseria gonorrhoeae lateral flow assay, *O-POCUS* Obstetric Point-of-care Ultrasound, *PMTCT* Prevention of mother to child transmission, *QA/QC* Quality Assurance and Quality Control, *RST* rapid syphilis test, *RDT* rapid diagnostic test, *TV* trichomonas vaginalis

**Table 5:** Inner setting factors influencing implementation of POC tests for maternal–infant screening and diagnosis

Reported barriers and facilitators	Associated studies and point-of-care interventions	Number of studies reporting factor	% of included studies reporting factor (*N* = 40)
*Facility readiness and infrastructure*			
Barriers			
Low facility readiness	• RST [75, 87, 92, 93, 95, 101] • HIV EID/birth testing [90, 97]; • Bundled PMTCT screening (HIV, syphilis, HBV) [79, 88]; • Dual HIV/Syphilis rapid test [82]; maternal HIV viral load [97]; • ABO blood group and Rh (rhesus) type [99]; • G6PD screening [100]; • O-POCUS [81]; • Bundled screening for HIV, syphilis, malaria, and anemia [105]	15	37.5%
Space constraints	• HIV EID/birth testing [83, 85, 86, 103] • Bundled PMTCT screening (HIV, syphilis, HBV) [79, 88] • mRDT [77] • HIV RDT [69]	8	20%
Unreliable power supply	• STI screening (GeneXpert CT/NG and TV Assays) [96]; • Bundled PMTCT screening (HIV, syphilis, HBV) [88]; • RST [92]; • O-POCUS [81]	4	10%
Facilitators			
Not reported		0	0%
*Resource availability*			
Barriers			
Stock shortages	• RST [67, 70, 75, 87, 92, 93, 98, 101]; • Bundled PMTCT screening (HIV, syphilis, HBV) [79, 88, 102]; • mRDT [76, 77, 98] • Anemia screening [70, 76, 98]; • Bundled screening for HIV, syphilis, malaria, and anemia [84, 105]; • HIV RDT [68, 69]; • HIV EID/birth testing [89, 90]; • Dual HIV/Syphilis rapid test [82] • Sickling, hepatitis C, blood type, and tuberculosis [98]; • O-POCUS [81]	21	52.5%
Human resource shortages	• HIV EID/BT PCR [80, 83, 89, 90]; • Bundled PMTCT screening (HIV, syphilis, HBV) [79, 88, 102]; • RST [75, 92] • Bundled screening for HIV, syphilis, malaria, and anemia [84, 105] • HIV RDT [69] • O-POCUS [81] • Maternal HIV viral load [91];	14	35%
Insufficient number of testing devices within facility	• HIV EID/birth testing [80, 83, 90]	3	7.5%
Facilitators			
Strong procurement planning	• RST [92, 94]; • HIV EID/birth testing [89];	3	7.5%
*Workflow integration and compatibility*			
Barriers			
Workload burden	• RST [67, 70, 75, 87] • HIV EID/birth testing [80, 83, 103]; • Bundled PMTCT screening (HIV, syphilis, HBV) [79, 88]; • Integrated diagnostic and clinical decision support system (CDSS) for POC anemia, preeclampsia, and gestational diabetes screening [78]; • HIV RDT [68–70]; • Anemia screening [70]; • mRDT [77]; • O-POCUS [81]; • Maternal HIV viral load [91];	15	37.5%
Poor workforce utilization and limited testing hours	• HIV EID/birth testing [89, 97]; • mRDT [77]; • Bundled screening for HIV, syphilis, malaria, and anemia [104]; • HIV RDT [69]; • Maternal HIV viral load [97];	5	12.5%
Lack of follow-up mechanisms for tracking clients who did not wait for test results	• HIV EID/birth testing [80, 89]; • HIV RDT [68]; • Bundled PMTCT screening (HIV, syphilis, HBV) [79]	4	10%
Facilitators			
Compatibility with existing clinic workflow	• HIV EID/birth testing [90, 97]; • Anemia screening [73, 76]; • Maternal HIV viral load [97]; • Dual HIV/Syphilis rapid test [82]; • mRDT [76] • RST [73] • HIV CD4 cell count [73];	5	12.5%
Optimizing layout and workflow scheduling	• Integrated diagnostic and clinical decision support system (CDSS) for POC anemia, preeclampsia, and gestational diabetes screening [78]; • HIV EID/birth testing [89]; • Bundled screening for HIV, syphilis, malaria, and anemia [84]	3	7.5%
*Training, skills, and competency*			
Barriers			
Inadequate training	• RST [67, 75, 87, 92, 93]; • mRDT [76, 77] • Bundled PMTCT screening (HIV, syphilis, HBV) [79, 88] • Anemia screening [76]; • HIV EID/birth testing [80]; • Bundled screening for HIV, syphilis, malaria, and anemia [84]	11	27.5%
Facilitators			
Cross-cadre training	• RST [67, 75, 94, 101]; • Bundled PMTCT screening (HIV, syphilis, HBV) [88]; • HIV EID/birth testing [89]; • O-POCUS [81^]^	7	17.5%
Refresher training	• HIV EID/birth testing [90]; • O-POCUS [81]; • Bundled PMTCT screening (HIV, syphilis, HBV) [102]	3	7.5%
Provider incentives	• RST [75]	1	2.5%
*Guideline dissemination and de-implementation*			
Barriers			
Poor dissemination of revised testing guidelines and algorithms	• RST [71, 75, 87, 92, 93]; • mRDT [76, 77]; • Dual HIV/syphilis rapid test [107]; • Anemia screening [76]	6	15%
Conflicting testing guidelines	• mRDT [77] • RST [92] • Maternal HIV viral load [91]; • HIV EID/birth testing [80]	4	10%
Lack of job aids for testing procedures	• HIV RDT [69]; • Bundled PMTCT screening (HIV, syphilis, HBV) [88]	2	5%
Facilitators			
Locally relevant reminder materials displayed within facility	• RST [92, 94];	2	5%
*Implementation climate and organizational culture*			
Barriers			
Poor cross-cadre communication	• mRDT and anemia screening [76];	1	2.5%
Facilitators			
Peer support and collaboration	• HIV EID/birth testing [80, 90]; • Bundled PMTCT screening (HIV, syphilis, HBV) [88]; • RST [92]; • O-POCUS [81];	5	12.5%
Strong facility leadership support	• RST [94]; • HIV EID/birth testing [90]	2	5%
Organizational agreement on intervention necessity and benefits	• HIV RDT [69]; • HIV EID/birth testing [86]	2	5%

*CT *chlamydia trachomatis, *EID* early infant diagnosis, *HBV* hepatitis B virus, *mRDT* malaria rapid diagnostic test, *NG* Neisseria gonorrhoeae, *NG-LFA* Neisseria gonorrhoeae lateral flow assay, *O-POCUS* Obstetric Point-of-care Ultrasound, *PMTCT* Prevention of mother to child transmission, *QA/QC* Quality Assurance and Quality Control, *RST* rapid syphilis test, *RDT* rapid diagnostic test, *TV* Trichomonas vaginalis

**Table 6:** Individual characteristics influencing implementation of POC tests for maternal–infant screening and diagnosis

Reported barriers and facilitators	Associated studies and point-of-care interventions	Number of studies reporting factor	% of included studies reporting factor (*N* = 40)
*Knowledge, skills, confidence*			
Barriers			
Low decision-making power among pregnant women/mothers	• Bundled PMTCT screening (HIV, syphilis, HBV) [79, 102]; • HIV EID/birth testing [83, 86]; • Bundled screening for HIV, syphilis, malaria, and anemia [84, 105]; • RST [92];	7	17.5%
Provider knowledge gaps	• RST [70, 75, 92]; • mRDT [77]; • HIV EID/birth testing [83]; • Anemia screening [70]; • Bundled screening for HIV, syphilis, malaria, and anemia [105]	6	15%
Limited engagement of client and partners in testing process	• RST [67, 72]; • Integrated diagnostic and clinical decision support system (CDSS) for POC anemia, preeclampsia, and gestational diabetes screening [78]; • Bundled PMTCT screening (HIV, syphilis, HBV) [79]; • HIV EID/birth testing [80]; • Bundled screening for HIV, syphilis, malaria, and anemia [84]	5	12.5%
Fear of result notification (from providers to patients and from patients to partners)	• Bundled PMTCT screening (HIV, syphilis, HBV) [79, 102]; • RST [92]; • Maternal HIV viral load [91]; • HIV EID/birth testing [83]; • Bundled screening for HIV, syphilis, malaria, and anemia [84];	5	12.5%
Limited knowledge among clients of disease risks and intervention benefits	• RST [72, 92, 95]; • Bundled PMTCT screening (HIV, syphilis, HBV) [79];	4	10%
Provider preference for established practices	• mRDT [76, 77]; • RST [92]; • Anemia screening [70];	3	7.5%
Facilitators			
Provider self-efficacy	• HIV EID/birth testing [90]; • RST [75]; • Bundled PMTCT screening (HIV, syphilis, HBV) [88]; • Dual HIV/syphilis rapid test [82]; • O-POCUS [81]	5	12.5%
Engagement of client and partners in the testing process	• mRDT and anemia screening [76]; • RST [101]; • Bundled PMTCT screening (HIV, syphilis, HBV) [88]; • HIV EID/birth testing [83];	4	10%
*Acceptance of the intervention*			
Barriers			
Hesitancy with test accuracy and procedures	• HIV EID/birth testing [80, 87, 90]; • mRDT [76, 77]; • Integrated diagnostic and clinical decision support system (CDSS) for POC anemia, preeclampsia, and gestational diabetes screening [78]; • RST [92]; • Anemia screening [76]; • Maternal HIV viral load [91];	8	20%
Low emotional preparedness for test result	• HIV EID/birth testing [83, 85, 86, 90]; • Bundled PMTCT screening (HIV, syphilis, HBV) [102]	5	12.5%
Unwillingness of pregnant women/ caregiver to wait for test result	• STI screening (GeneXpert CT/NG and TV Assays) [96]; • HIV EID/birth testing [89];	2	5%
Facilitators			
Trust in test accuracy	• HIV EID/birth testing [86, 90]; • RST [73]; • Anemia screening [73]; • Maternal HIV CD4 cell count [73]	3	7.5%
*Motivating factors*			
Barriers			
No barriers reported		0	0%
Facilitators			
Improved pregnancy and birth outcomes	• HIV EID/birth testing [80, 83, 85, 86, 89, 90]; • RST [67, 72, 87, 92]; • Dual HIV/syphilis rapid test [82]; • O-POCUS [81]; • mRDT and anemia screening [76]; • Maternal HIV viral load [91]; • Bundled PMTCT screening (HIV, syphilis, HBV) [79]; • Bundled screening for HIV, syphilis, malaria, and anemia [84]	16	40%
Accessibility to high quality health services	• HIV EID/birth testing [83, 85, 89, 90]; • Integrated diagnostic and clinical decision support system (CDSS) for POC anemia, preeclampsia, and gestational diabetes screening [78]; • O-POCUS [81]; • HIV RDT [68]; • mRDT and anemia screening [76];	8	20%
Health systems strengthening	• HIV EID/birth testing [80, 89]; • RST [87]; • Dual HIV/syphilis rapid test [107];	4	10%
Reduced caregiver anxiety	• HIV EID/birth testing [80, 83, 85, 86];	4	10%
Provider skill development and job satisfaction	• RST [75]; • HIV EID/birth testing [89]; • Bundled screening for HIV, syphilis, malaria, and anemia [84]	3	7.5%
*Patient-provider interpersonal relationship*			
Barrier			
Negative provider attitudes and power dynamic	• Bundled PMTCT screening (HIV, syphilis, HBV) [79]; • HIV EID/BT PCR [80]; • Bundled screening for HIV, syphilis, malaria, and anemia [84]; • mRDT and anemia screening [76];	4	10%
Facilitators			
Person-centred counselling and service delivery	• RST [75, 87, 95, 101]; • HIV EID/birth testing [80, 86, 89]; • Integrated diagnostic and clinical decision support system (CDSS) for POC anemia, preeclampsia, and gestational diabetes screening [78]; • HIV RDT [68]; • Maternal HIV viral load [91]; • Bundled PMTCT screening (HIV, syphilis, HBV) [79]; • Bundled screening for HIV, syphilis, malaria, and anemia [84]	12	30%
Patient trust in provider	• Integrated diagnostic and clinical decision support system (CDSS) for POC anemia, preeclampsia, and gestational diabetes screening [78]; • Bundled PMTCT screening (HIV, syphilis, HBV) [79]; • HIV EID/birth testing [83]	3	7.5%

*CT* chlamydia trachomatis, *EID* early infant diagnosis, *HBV* hepatitis B virus, *mRDT* malaria rapid diagnostic test, *NG* Neisseria gonorrhoeae, *NG-LFA* Neisseria gonorrhoeae lateral flow assay, *O-POCUS* Obstetric Point-of-care Ultrasound, *PMTCT* Prevention of mother to child transmission, *QA/QC* quality assurance and quality control, *RST* rapid syphilis test, *RDT* rapid diagnostic test, *TV* trichomonas vaginalis

**Table 7:** Process factors influencing implementation of POC tests for maternal–infant screening and diagnosis

Reported barriers and facilitators	Associated studies and point-of-care interventions	Number of studies reporting factor	% of included studies reporting factor (*N* = 40)
*Implementation within routine service delivery*			
Barriers			
Increased ANC consultation time and service demand pressure	• RST [67, 87]; • HIV EID/birth testing [89, 90]; • Integrated diagnostic and clinical decision support system (CDSS) for POC anemia, preeclampsia, and gestational diabetes screening [78]; • STI screening (GeneXpert CT/NG and TV Assays) [96]; • O-POCUS [81];	7	17.5%
Disruption to existing services	• Integrated diagnostic and clinical decision support system (CDSS) for POC anemia, preeclampsia, and gestational diabetes screening [78]; • RST, anemia, and HIV CD4 cell count [73]	2	5%
Facilitators			
Decentralized service delivery, including in community and home settings	• HIV EID/birth testing [80, 85, 89, 90]; • Bundled PMTCT screening (HIV, syphilis, HBV) [79, 102]; • RST [75]; • Dual HIV/syphilis rapid test [107]; • mRDT and anemia screening [76]; • Maternal HIV viral load [91]; • Bundled screening for HIV, syphilis, malaria, and anemia [84]	11	27.5%
Integration within existing programmes and workflows	• RST [67, 75, 94]; • HIV EID/birth testing [85, 103]; • Integrated diagnostic and clinical decision support system (CDSS) for POC anemia, preeclampsia, and gestational diabetes screening [78]; • Dual HIV/Syphilis rapid test [107]; • Bundled screening for HIV, syphilis, malaria, and anemia [84]	8	20%
Task-shifting	• O-POCUS [81]; • Dual HIV/syphilis rapid test [107]; • Maternal HIV viral load [91]; • HIV EID/birth testing [80]; • Bundled PMTCT screening (HIV, syphilis, HBV) [102]; • Bundled screening for HIV, syphilis, malaria, and anemia [105]	6	15%
Bundled testing	• RST [75, 87, 95]; • Bundled PMTCT screening (HIV, syphilis, HBV) [102]; • Bundled screening for HIV, syphilis, malaria, and anemia [105]	5	12.5%
*Quality assurance, control, and supervision*			
Barriers			
Lack of QA/QC processes and supervision	• RST [67, 75, 87, 92] • mRDT [77]; • Maternal HIV viral load [91];	6	15%
Facilitators			
Incorporation of QA/QC mechanisms from the outset of implementation	• RST [87, 94]; • HIV EID/birth testing [89, 90]; • HIV RDT [93]; • Bundled screening for HIV, syphilis, malaria, and anemia [105]	6	15%
Routine supportive supervision	• RST [67, 93]; • O-POCUS [81]; • Bundled PMTCT screening (HIV, syphilis, HBV) [102]; • Bundled screening for HIV, syphilis, malaria, and anemia [84]	5	12.5%
*Data, documentation, and surveillance systems*			
Barriers			
Inadequate data collection tools and surveillance systems	• RST [75]; • Bundled PMTCT screening (HIV, syphilis, HBV) [79]; • HIV EID/birth testing [90]; • Bundled screening for HIV, syphilis, malaria, and anemia [105]	4	10%
Documentation burden	• RST [67, 75];	2	5%
Facilitators			
Integrated surveillance	• Dual HIV/syphilis rapid test [107]; • HIV EID/birth testing [89]; • Maternal HIV viral load [91];	3	7.5%

*CT* chlamydia trachomatis, *EID* early infant diagnosis, *HBV* hepatitis B virus, *mRDT* malaria rapid diagnostic test, *NG* Neisseria gonorrhoeae, *NG-LFA* Neisseria gonorrhoeae lateral flow assay, *O-POCUS* Obstetric Point-of-care Ultrasound, *PMTCT* Prevention of mother to child transmission, *QA/QC* quality assurance and quality control, *RST* rapid syphilis test, *RDT* rapid diagnostic test, *TV* trichomonas vaginalis

### Intervention characteristics

Operational usability, design, reliability, cost, and relative advantage of POC tests strongly influenced implementation (Table 3).

Tests which were easy-to-use [67, 76, 84, 85, 87, 90, 92, 106], and which minimized procedural burden, such as single-finger-prick sampling [67, 72, 75, 84, 91, 102, 105] and simplified testing algorithms [72], supported operational adaptability and workflow integration across decentralized and high-volume settings. Conversely, complex procedures [82], disruptive sample collection [78], and intensive maintenance requirements [68, 89, 90], were identified as barriers. Tests with turnaround times exceeding 30 min, often further prolonged by resource constraints, contributed to retention challenges among pregnant women and caregivers [76, 80, 84, 86, 87, 89, 96, 97].

Design characteristics, such as portability [77, 81, 85, 87, 106], positive or negative interpretation [75, 87, 107], multiplex assays (dual HIV/syphilis tests) [82, 107], and discrete packaging [85], influenced adoption. Multi-disease platforms like GeneXpert (Cepheid, Sunnyvale, CA, USA) for POC maternal viral load and HIV EID were reported to streamline service delivery in high-volume settings [89, 91]; however, service delays due to competing demands for multiple tests on the same platform was also reported [91]. Tests which were difficult to interpret, such as faint positive lines, also hindered adoption [76, 84].

POC testing offered several advantages over centralized laboratory services across studies, including reduced follow-up costs, streamlined clinical workflows, and shorter turnaround times [72, 73, 76, 78, 81, 84–86, 88, 90, 90, 91]. Providers also felt that POC tests relieved pressure on health systems through reduced follow-up requirements and referrals to higher-level facilities [77, 81, 85, 87, 106].

Concerns over test validity, including false positive or negative results and device or operator errors [76, 77, 79, 91, 97, 103, 106], and technical issues such as cartridge defects, software failures, and power or internet disruptions [78, 80, 81, 85, 90, 92], undermined trust and delayed results, particularly in community settings.

While cost effectiveness [82, 91] and reduced indirect client costs [72, 78, 82, 85] were noted as key advantages, high test costs and limited affordability of treatment were commonly cited as barriers by providers and clients [75, 77, 79, 82, 89, 92].

### Outer setting

Implementation of POC tests was strongly shaped by the national and sub-national policy environment, funding, stakeholder engagement, supply chain and procurement systems, and community and household factors (Table 4).

Donor support was one of the most frequently reported external influences. Donor funding played a critical role in the initial rollout and ongoing maintenance of POC tests [67, 82, 87, 88]. While coordinated support from governments, donors, and implementing partners helped fill resource gaps and align guidelines [82, 90], heavy dependence on donor-funded screening—particularly for HIV—created implementation gaps for syphilis and HBV, limiting progress toward triple elimination goals [67, 69, 70, 79, 84, 86–88, 105]. Supportive national policies, including free testing services [67, 75, 91, 92, 107], and government-led policy and guideline dissemination [107] promoted implementation fidelity and sustainability. However, low prioritization in national policies, particularly for conditions outside major donor focus, along with vertical policy structures and narrow regulatory frameworks, were reported to hinder integration and coverage [77, 79, 80, 92, 102, 107]. Competing maternal-child health programme priorities and decentralized budgeting further constrained funding for syphilis and HBV screening [79, 88]. Poor stakeholder coordination [75, 77, 89], including the exclusion of frontline providers from implementation planning and decision-making [88], and hindered facility-level ownership.

Supply chain factors were also influential. Integration with existing prevention of vertical transmission supply chains and procurement processes facilitated implementation of dual HIV/syphilis and EID POC tests in two studies [82, 90]. However, disruptions were widely reported, especially for syphilis and HBV test kits and benzathine penicillin treatment for syphilis [67, 68, 70, 71, 75–77, 79, 84, 87–93, 102, 104]. These disruptions were often linked to fragmented procurement systems, low prioritization in national strategies, vertical funding streams, and inadequate forecasting [67, 70, 71, 76, 77, 79, 81, 82, 87, 88, 93, 98, 99, 101, 104].

Geographic, community, and family dynamics influenced POC test uptake. Community outreach and sensitization [86, 102, 107], male partner involvement [79, 80, 83], and household support [80] fostered trust, reduced misconceptions, and increased uptake of testing. In contrast, stigma—particularly related to HIV, syphilis, and other STIs—along with negative family and community influence, restrictive household decision-making, and entrenched gender norms, discouraged ANC attendance and acceptance of POC testing [70, 72, 79, 80, 84, 85, 92]. Other persistent barriers included late or missed ANC attendance in several communities [70, 80, 84, 92, 107], as well as geographic challenges and limited transportation, which restricted timely diagnosis and follow-up [68, 86, 88, 90, 92, 93, 98, 99].

### Inner setting

Within health facilities, facility readiness, resource availability, combability with workflows, training, guideline dissemination, and organizational climate shaped implementation (Table 5).

Facility readiness emerged as a central determinant of POC test implementation and sustainability. Multiple studies reported lower facility readiness in lower-level and rural facilities compared to urban or tertiary settings [79, 93, 95, 98, 99, 101, 105]. Facility readiness was also widely reported to be lower in private sector health facilities when compared to public sector [75, 92, 93, 99, 99, 100], and for the delivery of antenatal syphilis and other STI screening when compared to HIV [93]. Facility space constraints [69, 77, 79, 85, 88, 96, 103], and unreliable power supply [81, 88, 96] were also key barriers to adoption and sustained use.

Readiness was often linked to resource availability. Frequent stockouts and expired test kits [69, 75–77, 81, 82, 84, 88, 90, 92, 93, 99, 101] and insufficient numbers of POC devices at facilities [80, 86, 90, 98, 101] led to service disruptions, backlogs, unequal coverage, and increased wait times. Human resource shortages [69, 75, 79–81, 83, 84, 88, 91, 92], and high staff turnover [90, 102, 104], were also widely reported to impact adoption and sustained high-quality service delivery.

Integrating POC testing into established facility service delivery workflows improved feasibility [73, 76, 82, 90, 92, 97], however poor compatibility with existing resources, infrastructure, and patient volume created bottlenecks and service disruptions [79, 87, 97, 99] and lack of follow-up mechanisms [68, 80, 89] further hindered continuity of care. While one study reported that providers perceived POC EID tests to streamline and reduce workload [89], workload burden widely hindered uptake and service delivery continuity [67–70, 75, 77–81, 83, 87, 88, 91, 103], and was often linked to limited service hours and staff underutilization [69, 77, 89, 97, 104].

Several studies, however, noted that workload concerns diminished after implementation once workflows stabilized. For example, one evaluation reported that although most providers anticipated increased workload during the pilot of integrated POC testing for syphilis, anemia and CD4 + T-Cell count, only 21% reported this concern post-national introduction [73]. Another study found that adding rapid syphilis testing resulted in only a three-minute increase in consultation time [67], and modelling work suggested that integrated HIV, syphilis, malaria, and anemia screening could be accommodated within existing staff capacity [104].

Training quality, consistency, and access to information were key influencers of implementation. Hands-on, facility-based training [67, 81, 87–89], regular refresher training [81, 82, 102] enhanced organizational competency and service quality. In contrast, inadequate or inconsistent trainings [75–77, 80, 81, 84, 87, 92, 93, 99], and limited retraining opportunities [67, 75, 79, 80, 88, 90, 92] weakened organizational uptake and implementation fidelity.

Availability of context-adapted job-aids, such as instructions and informational material [92, 94], facilitated consistent use of POC tests. However, poor dissemination and de-implementation of revised testing guidelines and algorithms [71, 75, 76, 87, 92, 93, 107], conflicting testing protocols [77, 80, 91, 92], and lack of job aids [69, 88] undermined organizational uptake and implementation fidelity, particularly for syphilis and HBV screening.

Organizational culture played a dual role. Strong leadership, cohesive teamwork, and cross-cadre communication facilitated uptake [81, 88]. Organizational engagement [80, 86], awareness of the benefits of POC testing [69, 75, 80, 88, 90], and peer leader models [88, 92] further helped build buy-in and align services with broader maternal and neonatal health goals. However, poor facility-level coordination, unclear roles, and mistrust between teams undermined implementation [76, 77, 79, 89].

### Characteristics of individuals

Across providers, pregnant women, and caregivers, knowledge and confidence, acceptance of POC testing, motivational factors, and the quality of patient–provider relationships all shaped uptake and delivery of POC interventions (Table 6).

Healthcare providers viewed POC tests as filling critical gaps in maternal and newborn screening [67, 76, 80–85, 87–90], improving care quality [68, 76, 81, 88–90, 101] streamlining workflows [67, 80, 81, 86–90, 101], and strengthening the health care system more broadly [80, 81, 87–89, 98, 107], with increased job satisfaction [89], a sense of professional duty [75], and improved technical skills [84] drivers of adoption. Strong self-efficacy in performing tests, interpreting results, and acting on findings supported uptake [81, 82, 88, 90]. Confidence in test accuracy strengthened motivation [73, 90], but hesitancy arose when tests were perceived as unreliable [76, 85, 90, 91] or when providers were uncomfortable delivering sensitive results [79, 102]. Some providers expressed reluctance to abandon established practices. In the case of malaria RDT, this was due to concerns over missed diagnoses [76, 77], and in the case of rapid syphilis testing, providers perceived lab-based testing to enable comprehensive care by requesting women to return for treatment with their partner(s) [92]. Knowledge gaps, particularly for syphilis and HBV screening and treatment protocols, further led to testing errors and missed treatment opportunities [70, 75–77, 83, 92, 105].

Pregnant women and caregivers valued POC tests for enabling timely screening, diagnosis, and immediate management [67, 78–80, 83, 91, 99], reducing travel burden [72, 78, 85], and improving access [78, 85]. Motivation was driven by the desire to protect maternal and infant health [67, 79, 83]. Active involvement of pregnant women and caregivers in POC testing and treatment was a key enabler of acceptance and adherence [76, 83, 100]. Person-centred counselling and health education [68, 75, 78, 80, 84, 86, 87, 89, 91, 99, 100], supportive provider attitudes [78, 79, 81], and trusting patient-provider relationships [78, 79, 81] were widely reported to build confidence, support informed decision-making, and encourage engagement in the testing process. However, women’s decision-making power was constrained by household [79, 86] and provider [76] power dynamics, including partner or family approval requirements [79, 86, 92]. Mistrust of test reliability [77, 78] and fear of test results and disclosure [79, 83–85, 91, 92, 102], especially for testing in non-private or home-based testing, remained persistent barriers. Low partner engagement was also widely reported to hinder uptake [67, 72, 79, 80, 84]. Disease misconceptions [72, 79, 92] and concerns around sample collection, particularly for EID [78–80, 85, 91], further contributed to hesitancy among pregnant women and caregivers.

### Process

Implementation and maintenance of POC tests were strongly influenced by how services were integrated and delivered, the strength of quality assurance and quality control systems, and the reliability of data documentation and surveillance processes (Table 7).

Integration of POC tests into existing maternal–child health programmes, particularly prevention of vertical transmission, was a widely reported facilitator [67, 75, 78, 85, 94, 103, 104, 107]. Bundled screening improved service efficiency, strengthened care continuity, and reduced delays at both facility [67, 75, 78, 94, 100, 102–105, 107] and community levels [85, 102]. However, some studies reported that integration disrupted workflows, lengthened ANC visits, and strained system capacity [67, 78, 86].

Extending integrated models to decentralized settings—including rural clinics, outreach services, and lower-level health facilities—expanded access for hard-to-reach populations [79, 80, 84, 89–91, 107]. Task-shifting and task-sharing were widely used to manage workload, extend service hours, and optimize human resource use [80, 87, 91, 102, 105, 107], while facility-level adaptations such as flexible scheduling and workflow adjustments [78, 84, 89] helped tailor implementation to local contexts. Differentiated service delivery models, particularly community health workers, were seen as central to expanding testing coverage and providing follow-up care in home and outreach settings [80, 85, 102].

Consistent quality assurance and control mechanisms, including supportive supervision [67, 81, 84, 87, 93, 102], proficiency testing and external quality assessments [84, 93, 105] and adherence to standard operating procedures [89], were widely reported to enable sustainability of high-quality POC screening and diagnostic programmes. Where quality assurance and control systems were absent, poorly monitored, or irregularly implemented, provider trust and service quality were undermined [75–77, 87, 90–92]. Disease-specific disparities were reported—Nepal maintained robust quality assurance and control for HIV but had limited oversight for syphilis testing [93]. In Burkina Faso, low staff awareness of quality assurance and control protocols for syphilis testing limited adherence [67], while in Zambia, scaling back from monthly donor-led supervision during the rapid syphilis testing pilot to quarterly government-led visits during national roll-out reduced provider confidence [87].

POC testing was also seen as an opportunity to strengthen surveillance and health information systems with several studies noting perceived improvements in data quality and programme monitoring [89–91]. However, documentation gaps persisted: registers often lacked fields for syphilis and HBV results and treatment initiation [75, 91, 105], and even when updated tools existed, inconsistent recording and documentation burden contributed to incomplete data [67, 75, 79, 87, 91, 105].

## Discussion

This review aimed to identify multi-level factors influencing the integration of POC tests for screening and diagnosis in low-resourced maternal-child health settings. We found that successful implementation of POC testing relies on enabling technologies, complementary workflows, supportive systems, and strong community linkages, while long-term sustainability is constrained by persistent health system, financing, and infrastructure challenges.

POC tests were consistently preferred over laboratory- and symptom-based methods, enabling accurate, timely, and decentralized screening and management, and supporting progress toward maternal–infant health and vertical transmission elimination goals. However, turnaround times exceeding 30 min—often driven by facility- and system-level constraints such as high patient volumes, limited machines, and stockouts—led to patient attrition and undermined the benefits of test-and-treat strategies. These findings are consistent with multi-country evidence from POC EID [109] and STI screening [96, 110], showing that even when same-day results accelerate treatment initiation, uptake remains low. However, studies from South Africa demonstrated over 90% same-day treatment uptake among pregnant women despite 90-min turnaround times [111], with facility-level variation found [112]. These findings suggest that waiting behaviours are highly context-dependent and underscore the need for individual and systems-level research on factors shaping willingness and ability to wait. This information is essential to inform context-specific, person-centred test-and-treat strategies during pregnancy and the newborn period when timely treatment is essential [7, 31, 113].

External factors significantly shaped the enabling environment for POC test integration in maternal-child health programmes. A key theme identified in our review was the impact of low policy prioritization and vertical donor programming on non-HIV tests. Limited domestic focus and donor support, especially for syphilis and HBV, were linked to fragmented supply chains, stockouts, and gaps in quality assurance and control and surveillance. These findings align to evidence showing that low political commitment and siloed funding contribute to persistently lower syphilis screening coverage in pregnancy compared to HIV [58]. While donor support remains vital in low-resource settings [114], it is often delivered through disease-specific mechanisms that may hinder broader integration and promote dependency [115]. Recent funding cuts to major initiatives such as United States President's Emergency Plan for AIDS Relief (PEPFAR) and United States Agency for International Development (USAID)-supported programmes have already disrupted essential service delivery for maternal-child health programmes in several countries [116, 117], exposing the vulnerability of systems reliant on external financing. As donor funding continues to decline and countries take on more responsibility, weak national planning risks major service disruptions [118]. These findings underscore the need for strengthening political commitment, integrating supply chains and monitoring frameworks, and expanding domestic financing to ensure sustainable and high-quality delivery.

Within the inner setting, facility readiness varied widely, with rural and lower-level facilities facing greater constraints. Despite being primary ANC providers for many women in LMICs [119], these facilities often lack the infrastructure for high-quality maternal services [120–124]. Private-sector facilities also showed low readiness at both facility and provider levels. This is consistent with evidence that maternal POC services are more limited in these settings when compared to public facilities [125–127] despite the private sector's key role in ANC delivery in LMICs [128]. These findings highlight the need for hyperlocal, cross-sectional readiness assessments to guide scale-up and implementation fidelity. Adaptable tools from other maternal-child health domains, such as PrEP [129] and immunization [130], offer practical models for supporting implementation planning and targeted investment.

Compatibility with existing resources also emerged as critical for effective implementation within inner settings. Most studies reported severe workforce shortages and provider workload burden, which has been widely documented across LMICs [131]. This highlights that introducing new interventions without additional personnel or incentives risks staff burnout and compromises sustainability. Context-adapted and differentiated service delivery models, such as task-shifting and community health worker-delivered testing, were key to expanding access while easing facility-level burden. These models have improved coverage and cost-effectiveness of vertical transmission of HIV screening [132–134], obstetric ultrasound [135], and maternal malaria diagnosis and treatment [136] in resource-limited settings. However, their success depends on clear policies and regulatory frameworks, defined roles, and continuous competency-based training [137]. We also identified reluctance to shift from traditional approaches and gaps in guideline knowledge among healthcare workers as key barriers to the consistent and high-quality delivery of new point-of-care tests. This underscores the importance of a deliberate de-implementation period to phase out outdated or conflicting protocols before introducing new testing programmes, ensuring consistency, accuracy, and provider confidence in service delivery.

Integrating POC testing into routine maternal-child health services supported both uptake and long-term sustainability. Previous studies have demonstrated that bundled screening during antenatal and postnatal care is cost-effective [138–140], facilitates coordinated resource use, training, and supervision [141], enhances testing and treatment coverage [87, 140, 142], and increases perceived value among policy-makers and providers [82]. Given the frequent co-occurrence of conditions, such as HIV, STIs, malaria, and anemia, during pregnancy [22, 143–145], combined screening at shared timepoints offers important clinical and public health benefits. Several promising strategies to integrate and optimize maternal and newborn HIV and syphilis screening and treatment have been identified [146]; however, implementation research is needed to inform POC test integration strategies which align with facility capacity and minimize disruption.

Provider-initiated counselling and health education, provider-patient trust, and person-centred care facilitated acceptance among pregnant women and caregivers, which is aligned to prior findings from South Africa and Kenya linking these factors to greater engagement as well as partner disclosure [147, 148]. However, despite women’s strong motivation to protect their health and that of their unborn/newborn children, misinformation about disease risk, stigma, fear of disclosure, limited partner involvement, and restrictive gender norms hindered uptake. Prior research has underscored the influential role of communities, families, and partners in maternal-child service utilization in many LMIC [149–151], highlighting the importance of person-centred approaches, community and family sensitization, and integration of gender and social considerations into implementation strategies.

This review has several limitations. Few grey literature were identified and only one was eligible for inclusion, which may reflect limited public reporting of programme evaluations for POC screening implementation in maternal and newborn health, particularly outside major donor-supported initiatives, or operational findings that are not formally documented or publicly accessible. The peer-reviewed evidence base was heavily concentrated on HIV and syphilis POC tests, with far fewer studies examining other high-burden maternal conditions such as curable STIs beyond syphilis (*Neisseria gonorrhoeae, Chlamydia trachomatis, Trichomonas vaginalis*), hepatitis B, anemia, malaria, or hypertensive disorders. This imbalance likely reflects longstanding donor priorities and vertical programming focused on HIV and elimination of vertical transmission, which may have limited attention and resources for implementing and systematically evaluating a broader range of maternal and neonatal POC tests. Most included studies were conducted in a small number of African countries, and several were pre-pilot or controlled evaluations, reducing generalizability to routine settings and other regions. While relevant studies may also have been missed due to terminology or database constraints, these gaps point to the need for more geographically diverse, longitudinal, and context-specific implementation research that examines how system capacity, workflow demands, and sociocultural dynamics influence the delivery and scale-up of maternal and newborn POC tests.

## Conclusions

To our knowledge, this is the first systematic review to synthesize factors influencing the adoption, implementation, and sustainability of POC screening and diagnosis for maternal-child health in low-resource settings using an implementation science framework. While POC tests are widely valued by providers, pregnant women, and caregivers, success depends on strong, well-integrated health systems. To achieve their full potential and advance goals such as the elimination of vertical transmission of HIV, syphilis, and HBV, POC test-and-treat strategies must be embedded in well-resourced, coordinated, and people-centred health systems, supported by strong domestic leadership, sustainable financing, and context-specific delivery models. Addressing evidence gaps is essential to inform equitable scale-up of a broader range of POC tests and to ensure that diagnostic innovations translate into improved outcomes for pregnant women and newborns in low-resourced settings.

## Supplementary Information

Supplementary Material 1.

Supplementary Material 2.

Supplementary Material 3.

Supplementary Material 4.

## Data Availability

All data generated or analyzed during this study are included in this published article and its supplementary information files.

## Abbreviations

ANC: Antenatal care
CFIR: Consolidated Framework for Implementation Research
CT: *Chlamydia trachomatis*
EID: Early infant diagnosis
G6PD: Glucose-6-phosphate dehydrogenase
HBsAg: Hepatitis B surface antigen
HBV: Hepatitis B virus
HIV: Human immunodeficiency virus
JBI: Joanna Briggs Institute
LMIC: Low- and middle-income country
MMR: Maternal mortality rate
mRDT: Malaria Rapid Diagnostic Test
NAAT: Nucleic acid amplification test
NG: *Neisseria* *gonorrhoeae*
NG-LFA: *Neisseria **gonorrhoeae* Lateral Flow Assay
O-POCUS: Obstetric point-of-care ultrasound
PEPFAR: United States President's Emergency Plan for AIDS Relief
POC: Point of care
POCT: Point of care test
PrEP: Pre-exposure prophylaxis
PMTCT: Prevention of mother to child transmission
PRISMA: Preferred Reporting Items for Systematic Reviews and Meta-Analyses
PROSPERO: International Prospective Register of Systematic Reviews
QA/QC: Quality Assurance and Quality Control
RDT: Rapid diagnostic test
RST: Rapid syphilis test
SDG: Sustainable Development Goal
STI: Sexually transmitted infection
TV: *Trichomonas* *vaginalis*
USAID: United States Agency for International Development
WHO: World Health Organization
