# Supplementary material for: Factors influencing implementation of point-of-care tests for maternal and newborn screening and diagnosis in low-resource settings: a systematic review
Source: BMC Glob Public Health. 2026 Apr 11;4:34. doi: 10.1186/s44263-026-00261-2 (PMC13069759; doi:10.1186/s44263-026-00261-2)
Supplement: Supplementary file 1 — Supplementary Material 1. [file 44263_2026_261_MOESM1_ESM.docx]

**Supplementary Material 1:**

**Point-of-Care Tests for Maternal and Newborn Screening and Diagnosis: A Systematic Review of Factors Influencing Implementation in Low-Resource Settings**

Natalie Grace Shaetonhodi, Lindsey de Vos, Peya Brock, Dvora Joseph Davey, Alex de Voux, Andrew Medina-Marino

Table of Contents

[Supplemental Text S1: Definitions of test types included in systematically reviewed studies 1](#_Toc215928319)

[Supplemental Text S2: Full Systematic Review Search Strategy 3](#_Toc215928320)

[Eligibility Criteria 3](#_Toc215928321)

[Search Strategy 4](#_Toc215928322)

[***Search Terms and Boolean Operators*** 5](#_Toc215928323)

[References 6](#_Toc215928324)

# Supplementary Text S1: Definitions of test types included in systematically reviewed studies

***Blood group and Rhesus typing kits:*** Card- or cassette-based agglutination tests that rapidly determine ABO blood group and Rhesus status using capillary blood at the point of care.^1^

***Dual HIV/syphilis rapid tests****:* Combined lateral flow assays that detect HIV antibodies and treponemal antibodies for syphilis using one finger-prick sample and deliver results within 20 minutes. This test is generally used as a screening test, with positive results for HIV and/or syphilis requiring laboratory-based confirmatory testing as per national testing algorithms.^2^

***G6PD point-of-care tests:*** Qualitative or quantitative tests that detect glucose-6-phosphate dehydrogenase deficiency from a finger-prick sample to guide safe malaria treatment.^3,4^

***GeneXpert CT/NG and TV Assays***: Cartridge-based molecular nucleic acid amplification tests that automate sample preparation, amplification, and detection of *Chlamydia trachomatis, Neisseria gonorrhoeae*, and *Trichomonas vaginalis* within a closed system.^3,5^

***Haemoglobin point-of-care tests:*** Portable devices that measure haemoglobin concentration from a finger-prick sample to screen for anaemia during antenatal care.^6^

***Hepatitis B surface antigen tests (HBsAg RDTs):*** Lateral flow tests that detect hepatitis B surface antigen in whole blood or serum and provide same-day screening results.^7^

***HIV rapid diagnostic tests (HIV RDTs):*** Single-use lateral flow tests that detect HIV antibodies from a finger-prick blood sample and provide results within 15–20 minutes without instrumentation.^5,8^

***HIV Birth Testing and Early Infant Diagnosis****:* Virological assays, such as qualitative PCR or isothermal nucleic acid tests, which detect HIV nucleic acids rather than maternal antibodies. These tests enable diagnosis in infants younger than 18 months and support rapid ART initiation at birth, 4–6 weeks, and other recommended timepoints as per national guidelines.^9^

***HIV CD4+ T-Cell Count****:* Immunological test that quantifies circulating CD4+ lymphocytes to assess immune function among people living with HIV.^10^

***HIV Viral Load:*** Quantitative molecular assay that measures HIV RNA in blood and is the preferred method for monitoring ART effectiveness and detecting treatment failure.^8^

***Lateral Flow Assay:*** A rapid immunochromatographic test that uses capillary migration along a nitrocellulose strip to detect target antigens or antibodies.

***Malaria rapid diagnostic tests (mRDTs):*** Lateral flow assays detecting malaria antigens from capillary blood, enabling rapid diagnosis without microscopy.^11^

***Molecular Nucleic Acid Amplification (NAAT) tests:*** Cartridge-based or portable nucleic acid amplification assays that detect pathogen DNA or RNA at or near the site of care.^12^

***Obstetric point-of-care ultrasound (O-POCUS):*** Portable, handheld ultrasound devices used to assess foetal position, gestational age, viability, and high-risk obstetric conditions in low-infrastructure settings.^13^

***Rapid Syphilis Tests (RST)*** Treponemal or non-treponemal lateral flow tests that detect syphilis antibodies from whole blood and provide results at the point of care. Rapid syphilis tests are generally used as the screening test, with positive results requiring laboratory confirmatory testing based on the traditional or reverse algorithm.^5,14^

# Supplementary Text S2: Full Systematic Review Search Strategy

## ****Eligibility Criteria****

***Study Design:***

- **Inclusion:**
  - Observational, randomized and quasi experimental intervention studies, modelling, qualitative and mixed methods studies, meta-analysis
  - Implementation/program evaluations, Field and facility evaluations, quality improvement studies
  - Additionally, grey literature sources, including WHO reports, guidelines, and conference abstracts, will be reviewed
- Exclusion**:**
  - Systematic reviews, scoping reviews

***Population (P)***

- **Inclusion:**
  - Studies including pregnant women attending ANC in low-resourced settings.
  - Studies including newborns or mother-baby pairs undergoing POCT in low-resourced settings.
  - Studies including healthcare providers, facility managers, or policy makers involved in POCT implementation for pregnant women or newborns.
  - Studies including health facilities or service delivery points which provide antenatal or postnatal care.
- **Exclusion:**
  - Studies focusing exclusively on non-antenatal and non-postnatal populations or high-resource settings.

***Intervention (I)***

- Inclusion:
  - Studies which implement or evaluate the implementation of established/evidence-based or novel/emerging POC technology for diagnostics in pregnancy and newborn care.
- Exclusion:
  - Studies which only evaluate laboratory-based or non-point-of-care testing or diagnostic interventions

***Comparison (C)***

- No explicit comparator, as focus is on implementation. However, where applicable, comparison with standard-of-care or pre-intervention implementation (including from pilot to scale-up).

***Outcomes (O)***

- **Inclusion:**
  - **Studies which report barriers, facilitators or enablers, and outcomes related to implementation of POCT for pregnant women and newborns.**
- **Exclusion:**
  - Studies which only report on diagnostic accuracy or effectiveness without evaluation or discussion of real-world (i.e., non-research setting) implementation considerations (barriers, facilitators, or health systems/public health outcomes).

## Search Strategy

***Databases:***

The following databases will be systematically searched to ensure comprehensive coverage of relevant studies:

- PubMed
- MEDLINE
- Scopus
- Google Scholar

***Search Terms and Boolean Operators***

A combination of Medical Subject Headings (MeSH) terms and free-text keywords were used to develop a comprehensive search query. Boolean operators (AND, OR, NOT) and truncation (wildcards, e.g., *POCT* or **"point-of-care test*"**) were applied.

***Population (P) – Pregnant women, newborns***

- “antenatal care” OR “prenatal care” OR “maternal health” OR “pregnant women” OR “pregnancy” OR “ANC”
- “newborn care” OR “neonatal care” OR “postnatal care” OR “PNC” OR “infant” OR “neonates”

***Intervention (I) – Point-of-care testing (POCT) for maternal and neonatal infections***

- “implementation” OR “roll-out” AND
- “point-of-care testing” OR “POCT” OR “rapid diagnostic tests” OR “rapid testing”

***Comparison (C) – No explicit comparator***

- No comparator search terms will be used, as this review focuses on implementation studies.

***Outcomes (O) – Barriers, facilitators, health system & public health outcomes***

- “barriers” OR “challenges” OR “obstacles” OR “limitations”
- “facilitators” OR “enablers” OR “drivers” OR “health system” OR “maternal health” OR “neonatal health” OR “testing coverage” OR “treatment coverage” OR “case detection” OR “treatment accuracy”

# References

1. Mitra R, Mishra N, Rath GP. Blood groups systems. *Indian Journal of Anaesthesia*. 2014;58(5):524-528. doi:10.4103/0019-5049.144645

2. World Health Organization. Dual HIV/syphilis rapid diagnostic tests. Global Sexually Transmitted Infections Programme. 2025. Accessed December 6, 2025. https://www.who.int/teams/global-hiv-hepatitis-and-stis-programmes/stis/testing-diagnostics/dual-hiv-syphilis-rapid-diagnostic-tests

3. World Health Organization. *Tests for Glucose-6-Phosphate Dehydrogenase Activity: Target Product Profiles*. 1st ed. World Health Organization; 2022. https://www.who.int/publications/i/item/9789240062962

4. World Health Organization. G6PD RDTs. Global Malaria Programme. 2025. Accessed December 6, 2025. https://www.who.int/teams/global-malaria-programme/case-management/diagnosis/rapid-diagnostic-tests/g6pd-rapid-diagnostic-tests

5. World Health Organization. *Laboratory and Point-Of-Care Diagnostic Testing for Sexually Transmitted Infections, Including HIV*. 1st ed. World Health Organization; 2023. https://www.who.int/publications/i/item/9789240077089

6. World Health Organization. *Haemoglobin Point of Care Analysers*. Vol 22. 1st ed.; 2025. https://www.who.int/publications/b/73511

7. Xiao Y, Thompson AJ, Howell J. Point-of-Care Tests for Hepatitis B: An Overview. *Cells*. 2020;9(10):2233. doi:10.3390/cells9102233

8. World Health Organization. *Consolidated Guidelines on Differentiated HIV Testing Services*. World Health Organization; 2024. https://www.who.int/publications/i/item/9789240096394

9. World Health Organization. *Updated Recommendations on HIV Prevention, Infant Diagnosis, Antiretroviral Initiation and Monitoring*. World Health Organization; 2021. https://www.who.int/publications/i/item/9789240022232

10. World Health Organization. *Point-of-Care CD4 Tests to Support the Identification of Individuals with Advanced HIV Disease*.; 2020. https://www.who.int/publications/i/item/point-of-care-test-for-identifying-people-living-with-advanced-HIV-disease

11. World Health Organization. The role of RDTs in malaria control. Global Malaria Programme. Accessed December 6, 2025. https://www.who.int/teams/global-malaria-programme/case-management/diagnosis/rapid-diagnostic-tests/role-in-malaria-control

12. Zu Y, Chang H, Cui Z. Molecular point-of-care testing technologies: Current status and challenges. *Nexus*. 2025;2(2):100059. doi:10.1016/j.ynexs.2025.100059

13. Van Der Zande JA, Rijs K, Shamshirsaz AA, et al. The role of point-of-care ultrasound (POCUS) in maternal medicine. *Best Practice & Research Clinical Obstetrics & Gynaecology*. 2025;100:102599. doi:10.1016/j.bpobgyn.2025.102599

14. World Health Organization. *WHO Guideline on Syphilis Screening and Treatment for Pregnant Women*. World Health Organization; 2017. https://www.who.int/publications/i/item/9789241550093
